# State Medical Convention

**Published:** 1848-05

**Authors:** 


### RECORD OF MEDICAL SCIENCE.

### STATE MEDICAL CONVENTION.

We extract the following summary of the proceedings of the late Medical Convention, held at Lancaster, Pa., from the "Lancaster County Farmer," the Editor of which paper was present, by authority of the Convention, for the purpose of reporting its transactions.

In pursuance of a call published in this and other papers throughout the State, this body convened in the Methodist Church in this city (Lancaster,) on Tuesday last, at eleven o'clock, A. M., and on motion of Dr. Geo. B. Kerfoot, of this city, Dr. Heister of Berks county, was called to the Chair, and Dr. Stillé of Philadelphia, appointed Secretary *pro tem.*

Motion by Dr. Kerfoot—that A. G. Williams be permitted to occupy a seat in the Convention and report proceedings for publication—carried.

A committee was appointed to examine the credentials of delegates and report to the Convention. After these had been presented, the committee reported through their Chairman, Dr. John L. Atlee, the following persons as entitled to seats in the Convention.

*Jefferson Medical College*—Drs. Thomas D. Mütter, R. M. Huston.

*College of Physicians*—Drs. Joseph Carson, Lewis Rodman, Saml. Jackson, Isaac Hays, A. Stillé, F. G. Smith, George Fox, V. L. Godon, W. B. Page, W. L. Atlee.

*Philadelphia Medical Society*—Drs. George W. Norris, Henry H. Smith, John Bell, B. H. Coates, Isaac Parrish, G. Emerson, Henry Bond, J. R. Paul, M. Clymer, E. Hartshorne.

*Pennsylvania Medical College*—Drs. Henry S. Patterson, W. R. Grant.

*Franklin Medical College*—Drs. Thomas F. Betton, P. B. Goddard.

*Philadelphia Association for Medical Instruction*—Dr. Francis West.

*Phila. College of Medicine*—Drs. H. Gibbons, J. B. Burden.

*Northern Medical Society of Phila.*—Drs. James W. Stewart, Wm. Mayberry, J. F. Lamb, A. Helfenstein, Thos. Hobson, John Rhein.

*Lancaster Co. Medical Society*—Drs. J. L. Atlee, Saml. Humes, Geo. B. Kerfoot, Samuel Duffield, J. S. Clarkson, J. K. Eshelman.

*Lancaster Co. Hospital*—Drs. H. A. Smith, C. O. Richards.

*York Co. Medical Association*—Dr. E. S. Shearer.

*Schuylkill Co. Medical Association*—Drs. James S. Carpenter, J. G. Kœhler, J. S. Morton Zulick.

*Chester Co. Medical Society*—Drs. W. W. Townsend, Samuel H. Harry, C. W. Parrish and E. F. Rivinus.

*University of Pennsylvania*—Drs. S. Jackson, W. Gibson.

*Lycoming Co.*—Dr. Thomas Wood.

*Lebanon Co. Medical Society*—Drs. D. B. Marshall, Samuel Behm, B. L. Schenk.

*Medical Faculty of Berks Co.*—Drs. Jno. P. Hiester, W. Moore, J. H. Seltzer.

*Bucks Co.*—Dr. C. W. Foulke.

*Susquehanna Co.*—Dr. G. S. Dimock.

*Dauphin Co.*—Drs. E. Roberts, A. Miller.

It was then moved by Dr. J. L. Atlee, that Medical gentlemen present from the counties not otherwise represented, be invited to take seats, and participate in the deliberations of the Convention. Carried.

Moved by Dr. Mütter, that Dr. F. A. Muhlenberg be invited to a seat in the Convention, with all the privileges of membership. Carried.

Dr. Emerson, of the Philadelphia Medical Society, offered the following preamble and resolution:

Whereas, The extension of knowledge upon all subjects pertaining to the Healing Art, and the improvement of the capacities of those to whose skill and attention the suffering community is necessarily entrusted, are matters of the deepest interest to the public. Therefore, we, the Representatives of the Medical Faculty of the State of Pennsylvania, believing that these objects may be greatly promoted by a systematic organization of the members of the profession of the Commonwealth, assembled in Convention, in the city of Lancaster, do

*Resolve*, That we now proceed to the organization of a State Medical Society. Passed.

On motion, a committee was appointed to nominate officers of the Convention, (names of the gentlemen composing this Committee, were not distinctly heard by the reporter.) After a short consultation the Committee reported as follows:

*For President*—Dr. Samuel Humes, of Lancaster county.

*For Vice Presidents*—Dr. Heister, of Berks county, Dr. Wood, of Lycoming.

*For Secretaries*—Dr. Stillé, of Philadelphia, Dr. Dimock, of Susquehanna.

Report accepted and nominations of the committee confirmed by the Convention.

Dr. Humes was then conducted to the Chair by Dr. Heister, President pro tem. and expressed his gratitude to the Convention for the honor conferred upon him, in a short and appropriate address.

Upon taking his seat, Dr. Stillé asked to be excused from serving as Secretary of the Convention. On motion he was excused, and the name of Dr. Marshall, of Lebanon county, substituted.

The object for which the Convention was called, was then formally stated to be the organization of a State Medical Society.

Moved by Dr. Emerson, that a committee be appointed for the purpose of drafting and presenting to the Convention a Constitution, under which the organization of a State Society might be effected. The Committee was composed of the following gentlemen, viz: Drs. Hays, J. L. Atlee, Emerson, Bond, Betton, Duffield, Eshelman and Harris.

The Convention then adjourned till 3½ o'clock, in the afternoon.

—

3½ o'clock, P. M.

Met pursuant to adjournment. Several Delegates, not present at the organization, appeared, and took their seats.

On motion, the roll was called.

On motion, Dr. Francis Burrows of Lancaster, was admitted to a seat in the Convention.

Report of the committee to draft a Constitution was called for, when it was presented by Dr. Hays, as Chairman of that committee, read and accepted.

Motion by Dr. J. L. Atlee, that Hon. Judge Lewis be invited to take part in the proceedings of the Convention—motion prevailed, and a committee was appointed to wait on and conduct the honorable gentleman to a seat.

It was moved that the Convention now proceed to take up, and discuss and adopt the Constitution,—(by the separate article.) Carried.

Considerable debate was had upon each article of the Constitution, enlivened by occasional sallies of wit, &c., nevertheless, harmony prevailed throughout the proceedings.

On motion, the Convention adjourned till 8 o'clock on Wednesday morning.

*Wednesday*, April 12.

Convention met pursuant to the adjournment of last evening. Dr. Hays moved that the consideration of the Constitution be resumed, which being done, an animated discussion ensued, in which, Drs. Bond, Hays, Carpenter, Parrish, Judge Lewis and others participated.

The following Constitution was finally agreed to, by the Convention.

ARTICLE I.—*Title of the Society.*

The name and title of this Society shall be "The Medical Society of the State of Pennsylvania."

ARTICLE II.—*Objects of the Society.*

The objects of this Society shall be the advancement of Medical knowledge, the elevation of professional character, the protection of the interests of its members, the extension of the bounds of medical science and the promotion of all measures adapted to the relief of suffering, and to improve the health and protect the lives of the community.

ARTICLE III.—*Members of the Society.*

§ 1. The Society shall consist of Delegates and Associates.

§ 2. The delegates shall receive their appointment from the County Societies.

§ 3. Every delegate before admission to a seat in the Society, shall produce a certificate of delegation signed by the President or Secretary of his County Society, shall sign the Constitution, and shall pay the assessment.

§ 4. Each delegate shall hold his appointment for one year, or until another is appointed to succeed him.

§ 5. Every member of a County Society shall be an associate of the State Society so long as he conforms to its regulations.

ARTICLE IV.—*Of the Officers.*

§ 1. The officers of the Society shall be a President, four Vice Presidents, a corresponding Secretary, two recording Secretaries, a Treasurer, and five Censors for each of the six Censorial Districts.

§ 2. Each officer shall be elected by vote, on a general ticket, and shall serve for one year, or until another is elected to succeed him.

§ 3. None but delegates shall be eligible to the offices of President, Vice President, Secretaries, or Treasurer; but delegates and associates, provided they have been fifteen years in practice, shall be eligible to the office of Censor.

ARTICLE V.—*Duties of officers.*

§ 1. The President shall preside at the meetings, preserve order, and perform such other duties as custom and parliamentary usage may require. He shall not be eligible two terms in succession.

§ 2. The Vice President, when called upon, shall assist the President in the performance of his duties, and during the absence of, or at the request of the President, one of them shall officiate in his place. They shall not be eligible two terms in succession.

§ 3. The Corresponding Secretary shall conduct the correspondence and perform such other duties as usually appertain to that office.

§ 4. The Recording Secretaries shall keep correct minutes of the proceedings of the Society, and, when approved of, shall fairly transcribe the same in a book to be kept for the purpose. They shall have charge of all papers belonging to the Society, other than those appertaining to the Treasurer and Corresponding Secretary, and shall give due notice of the annual meetings.

§ 5. The Treasurer shall receive all monies belonging to the Society and disburse them as directed, preserving vouchers for the same. He shall annually present a statement of the finances of the Society; which shall be referred to a committee of three delegates to be audited. He shall give security for the faithful performance of his duties, whenever the council shall judge it requisite.

§ 6. It shall be the duty of the Censors of each District to examine the laws and regulations of the County Societies, and if they find nothing in the said laws and regulations contrary to the letter or spirit of those of the State Society, the Censors shall endorse on them the word "approved," with their signatures, and the date of such approval, and transmit one copy to the corresponding Secretary of the State Society and another to Secretary of the County Society.

§ 7. It shall also be their duty in case of appeal from the decision of a County Society by a member, who may conceive himself aggrieved thereby, to examine into the merits of the case, and give their decision which shall be final. They shall report their decisions through the corresponding Secretary, annually.

§ 8. Three censors shall constitute a quorum, and be competent to transact business.

Article VI.—*Of the County Societies.*

§ 1. The members of the Profession in any county of this State, who desire so to do, may form themselves into a county society, provided, that public notice of the proposed meeting for the purpose be given, that all the regular members of the Profession in the county be invited to join therein; and said Society may adopt rules for their government, provided the same do not contravene those of the State Society, may elect officers and do such other matters as may be necessary to carry out the objects of their Association.

§ 2. No one shall be admitted as a member of a county Society unless he is either a graduate in medicine of some respectable medical school, or has a license to practice from a Board recognized by the State Society, or has been a practitioner for at least fifteen years, and who, moreover, is in good moral and professional standing in the place where he resides.

§ 3. Any physician who shall procure a patent for a remedy, or instrument of surgery, or who sells or deals in patent remedies, or nostrums, or who prescribes a remedy without knowing its composition, or who shall hereafter give a certificate in favor of a patent remedy, or instrument, shall be disqualified from becoming a member of a County Society.

§ 4. As soon as a County Society is organized, the Secretary thereof shall transmit to the censors of the District, two copies of their rules and regulations with the names of the officers and members, and as soon as one of the said copies is returned with the approval of the said censors or a majority of them, the society shall be authorized to elect one delegate to the State Society for every ten of its members, and one Delegate where the Society does not consist of ten members.

§ 5. Every County Society shall enforce the observance by its members of the code of Ethics adopted by the State Society, and they shall be authorized to censure or expel any members convicted of violating its provisions.

§ 6. A member of a County Society who is censured or expelled, shall have a right to appeal to the Censors of the District.

§ 7. A member who is expelled, shall be debarred from the rights of consultation, or the privileges of professional intercourse with any member of the State Society.

§ 8. The County Societies shall report annually to the State Society a list of their officers and members, any new rules which they may adopt, and such other matters as they may deem interesting.

§ 9. Each County Society shall have a right to fix a fee-bill for regulating the charges of its members for their Professional services.

§ 10. The County Societies shall hold, at least two meeting in each year.

§ 11. Each Society shall have full authority to adopt such measures as they may deem most efficient for mutual improvement, for exciting a spirit of emulation, for facilitating the dissemination of useful information, for promoting friendly intercourse among its members, and for the advancement of Medical Science.

§ 12. If any County Medical Society shall neglect to perform all such acts as may be required to be done by the laws of the State Society, or be guilty of any act which may be considered derogatory to the honor of the Medical Profession, or shall oppose or neglect to comply with the laws of the State Society, such County Society, during such delinquency, shall have their privileges, as a portion of the State Society, suspended, and their delegates shall not be entitled to a seat in the State Society.

### Article VII.—*Meetings of the Society.*

§ 1. The Society shall hold an annual meeting in the month of April, of each year.

§ 2. The time and place of meeting shall be determined for each succeeding year by a vote of the Society.

### Article VIII.—*Of the Funds.*

§ 1. Funds for defraying the expenses of the annual meetings, and the current expenses of the Society, may be raised by an assessment of the County Societies, of not more than two dollars for each delegate which said County Societies may be entitled to.

ARTICLE IX.—*Code of Ethics.*

§ 1. This Society adopts as a part of its regulations, the code of Ethics of the American Medical Association.

ARTICLE X.—*Of the Censorial Districts.*

§ 1. The State shall be divided into six Districts as follows, viz. The first shall comprise the counties of Philadelphia, Delaware, Chester, Montgomery, Bucks, Lehigh, Berks, Schuylkill, Lebanon, Dauphin and Lancaster.

The second, counties of Northampton, Pike, Wayne, Susquehanna, Luzerne, Columbia, Northumberland, Bradford.

The third of Lycoming, Tioga, Potter, Centre, Union.

The fourth of Mifflin, Huntingdon, Perry, Cumberland, Adams, York, Franklin, Bedford.

The fifth, counties of Beaver, Alleghany, Washington, Green, Fayette, Westmoreland, Cambria, Indiana, Armstrong, Butler.

The sixth of Mercer, Jefferson, Crawford, Erie, Warren, McKean, Clearfield, and such counties as have been, or may be formed of the above counties in each district.

ARTICLE XI.—*Provision for Amendments.*

Every proposal for altering or amending this Constitution, shall be made in writing; and if such alteration or amendment receive the unanimous vote of the members present, it shall be adopted; but, if objections be made, the alteration or amendment shall lay over until the next annual meeting, when, if it receive the vote of two-thirds of the voters present it shall be adopted.

BY LAWS.—*Of the Order of Business.*

§ 1. The President, or, in his absence, one of the Vice Presidents, shall call to order, or in case of the absence of all these officers, a Chairman shall be appointed pro tem. for the purpose.

§ 2. The appointment of a committee to examine the credentials of Delegates.

§ 3. The report of the above named committee.

§ 4. Calling the Roll.

§ 5. Election of Officers.

§ 6. Reading of the minutes.

§ 7. Any business which requires early consideration may, by permission, be introduced.

§ 8. Reports from the County Societies.

§ 9. The correspondence shall be read by the Corresponding Secretary.

§ 10. Written communications on Medical subjects may be read and discussed.

§ 11. Oral communications may be made and discussed.

§ 12. Resolutions introducing new business.

§ 13. The selection of a place for the next meeting of the Society.

§ 14. Unfinished and miscellaneous business.

§ 15. Adjournment.

On motion of Dr. Patterson, each member of the Convention contributed the sum of one dollar, to defray the expenses of printing the minutes to be published under the direction of the Lancaster County Medical Society.

On motion of Dr. Burden—

Resolved, That the thanks of this Convention be tendered to the Trustees of the Methodist Episcopal Church for the use of their house; and likewise to the Lancaster Co., Medical Society for their kindness and hospitality.

Dr. Kerfoot introduced the following Preamble and Resolutions, which were read:

WHEREAS, It is evident that, for want of some wholesome law to regulate and guard the practice of Medicine in the State of Pennsylvania, disqualified persons are permitted to impose themselves upon the public in undertaking what they do not understand, and pretending to what they do not possess, to the great injury of human health, and danger to human life: Therefore,

Resolved, That, as honest and scientific attainments are considered essential pre-requisites in every department of professional life, we deem them paramount in the assumption of medical character.

*Resolved*, That, as the guardians of the health and lives of the citizens of this Commonwealth, we respectfully call the attention of Government and the people to the serious consideration of the establishment of a State Medical Tribunal, without whose authority, or that of an incorporated College, none shall practice, under penalty.

*Resolved*, That a bill can be draughted, and may become a law, which will be in keeping with the age, compatible with the spirit of our Republican institutions, and meet the wants of the people.

*Resolved*, That a committee of five be appointed to frame a bill which shall be submitted to the next annual meeting of the Medical Society of the State of Pennsylvania, and, if approved, shall then be presented to the next Legislative body for their consideration.

These resolutions occasioned much desultory debate; and on motion of Dr. Carpenter of Pottsville, were laid on the table for the time being.

Dr. Stillé offered the following resolutions:

1st. That all unfinished business of the Convention be referred to the Medical Society of the State of Pennsylvania, about to be organized:

2d. That the members of the Convention do now resolve themselves into a body under the style and title of "The Medical Society of the State of Pennsylvania."

3d. That the Officers of this Convention continue to act as Officers of the Society until the permanent organization of the Society—passed.

The Constitution, as approved in Convention, was then adopted by the Society.

On motion of Dr. Fox—

A Committee, consisting of one from each body represented, was appointed to nominate Officers of the Society for the ensuing year. The Committee retired to transact the business of their appointment.

On motion of Dr. Gibbons—

The Society proceeded to elect, by ballot, delegates to the American Medical Association. Drs. Gibbons and Page were appointed tellers. After counting the votes, the following named gentlemen were declared duly elected:

Drs. Jno. L. Atlee, of Lancaster; Worthington, of Chester; Jno. P. Heister of Berks: McIlvain of York; J. S. Carpenter, of of Schuylkill; Samuel Jackson, of Philadelphia.

Resolution by Dr. Grant—

*Resolved*, That the thanks of the Society be tendered to the President and other officers of the late State Medical Convention, for the able manner in which they have discharged their respective duties. Passed.

By Dr. Stillé—

*Resolved*, That the several County Medical Societies be requested to procure the registration of the names of all regular practitioners in their respective limits, and transmit a list of the same to the State Society, at the next meeting. Passed.

By. Dr. Parrish—

*Resolved*, That the Lancaster Co. Medical Society be instructed to transmit copies of the proceedings of the Convention and Society to the address of prominent physicians residing in those sections of the State, not here represented.

The Committee on nominations came in, and reported, through Dr. Jno. L. Atlee, as follows;

Dr. Samuel Humes, of Lancaster, *President*.

*Vice Presidents*—Drs. J. P. Heister, of Berks; Thomas Wood, of Lycoming; Prof. Sam'l Jackson, of Phila; and Jno. L. Atlee, of Lancaster.

*Corresponding Secretary*—Dr. Isaac Hays of Phila.

*Recording Secretaries*—Drs. H. S. Patterson, of Phila., George B. Kerfoot of Lancaster.

*Treasurer*—Dr. George Fox, of Philadelphia.

*Censors for South East District*—Drs. F. A. Muhlenberg of Lancaster, George W. Norris of Philadelphia, James S. Carpenter of Schuylkill, T. Hiram Corson of Montgomery, and Wilmer Worthington of West Chester.

Report accepted, and nominations confirmed.

Moved by Dr. Heister—

That, when the Society adjourn, it adjourn to meet at Reading on the 2d Tuesday in April, next.

Amendments, substituting other places, were offered, but lost.

Dr. Atlee moved to amend by substituting 2d Wednesday for 2d Tuesday. Carried.

The question recurring on the original motion of Dr. Heister, it was carried—fixing Reading as the place, and 2nd Wednesday in April, next, as the time of holding the next meeting.

On motion of Dr. Kerfoot—

*Resolved*, That this Society approves the standard of Medical Education recommended by the American Medical Association, and urges upon County Societies its strict observance. Passed.

Dr. Marshall called up the Preamble and Resolutions introduced in Convention by Dr. Kerfoot, and, on motion of Dr. Coates, they were ordered to lie over, and recommended to the early attention of the next annual meeting of the State Society.

On motion of Dr. Atlee, the Society adjourned without day.

